# Medicines reconciliation in comparison with NICE guidelines across secondary care mental health organisations

**DOI:** 10.1007/s11096-015-0236-7

**Published:** 2016-01-06

**Authors:** Medha Kothari, Ian Maidment, Ray Lyon, Lynn Haygarth

**Affiliations:** Aston University, Aston Triangle, Birmingham, B4 7ET UK; Sussex Partnership NHS Foundation Trust, Brighton, West Sussex BN13 3EP UK; University of Huddersfield, Huddersfield, West Yorkshire HD1 3DH UK

**Keywords:** Medication reconciliation, Mental health, Mental health organisations, Secondary care, United Kingdom

## Abstract

*Background* Medicines reconciliation—identifying and maintaining an accurate list of a patient’s current medications—should be undertaken at all transitions of care and available to all patients. *Objective* A self-completion web survey was conducted for chief pharmacists (or equivalent) to evaluate medicines reconciliation levels in secondary care mental health organisations. *Setting* The survey was sent to secondary care mental health organisations in England, Scotland, Northern Ireland and Wales. *Method* The survey was launched via Bristol Online Surveys. Quantitative data was analysed using descriptive statistics and qualitative data was collected through respondents free-text answers to specific questions. *Main outcomes measure* Investigate how medicines reconciliation is delivered, incorporate a clear description of the role of pharmacy staff and identify areas of concern. *Results* Forty-two (52 % response rate) surveys were completed. Thirty-seven (88.1 %) organisations have a formal policy for medicines reconciliation with defined steps. Results show that the pharmacy team (pharmacists and pharmacy technicians) are the main professionals involved in medicines reconciliation with a high rate of doctors also involved. Training procedures frequently include an induction by pharmacy for doctors whilst the pharmacy team are generally trained by another member of pharmacy. Mental health organisations estimate that nearly 80 % of medicines reconciliation is carried out within 24 h of admission. A full medicines reconciliation is not carried out on patient transfer between mental health wards; instead quicker and less exhaustive variations are implemented. 71.4 % of organisations estimate that pharmacy staff conduct daily medicine reconciliations for acute admission wards (Monday to Friday). However, only 38 % of organisations self-report to pharmacy reconciling patients’ medication for other teams that admit from primary care. *Conclusion* Most mental health organisations appear to be complying with NICE guidance on medicines reconciliation for their acute admission wards. However, medicines reconciliation is conducted less frequently on other units that admit from primary care and rarely completed on transfer when it significantly differs to that on admission. Formal training and competency assessments on medicines reconciliation should be considered as current training varies and adherence to best practice is questionable.

**Electronic supplementary material:**

The online version of this article (doi:10.1007/s11096-015-0236-7) contains supplementary material, which is available to authorized users.

## Impacts of findings on practice

- The majority of secondary care mental health organisations appear to be complying to NICE guidance as nearly 80 % of organisations are carrying out medicines reconciliation within 24 h of admission on acute wards.
- Medicines reconciliation on patients admitted via crisis and home treatment teams is an area where further research and guidelines are needed as only 38 % of organisations report carrying it out.
- Medicines reconciliation on transfer is currently much quicker and less exhaustive to the process on admission.
- Methods of staff training on medicines reconciliation currently differ between all trusts. There is doubt on its adherence to clinical excellence, as this research has shown there frequently are no formal training procedures put in place by the trusts.

## Introduction

Precise and trustworthy information about patient medication, including how well they are adhering to their medication regimen, must be obtained every time a patient is transferred from one healthcare setting to another [1]. This includes: names, dosages, frequencies, and routes of administration, allowing healthcare professionals to compare the patient’s previous medication list with their current medication list [2–4]. Timely and informed decisions can then be made regarding the patient’s future treatment and any discrepancies can be accounted for, documented and dealt with appropriately [1, 5]. This is the process of medicines reconciliation [1–5].

Accuracy and promptness in completing medicines reconciliation decreases the risk of medication errors being made in primary and secondary care, when transferring across these boundaries as well as when transferring within them [4]. In 2007, Medicines Reconciliation guidelines were published by The National Institute for Health and Care Excellence (NICE) in collaboration with the National Patient Safety Agency (NPSA), identifying the aim of the process: to confirm that medicines prescribed upon admission correlate to those taken by the patient prior to admission in order to reduce adverse drug events [4, 5]. More recent NICE guidance [6] and World Health Organisation (WHO) guidance [7] suggest medicines reconciliation should be completed within 24 h of admission. The recent NICE guidance also identified that the process may need to be carried out more than once during the admission, for example on transfer between wards [6]. Additionally all relevant information must be documented on paper or electronically [6].

In this study, a national survey on the practise of medicines reconciliation in mental health organisations was completed by chief pharmacists or those with equivalent status. The survey was specifically sent out to members of the College of Mental Health Pharmacy (CMHP), a UK organisation dedicated to advancing education and promoting research in the practice of mental health pharmacy for the benefit of the public.

Traditionally, junior doctors have had the responsibility of undertaking medicines reconciliation, increasingly this is becoming a pharmacist and pharmacy technician’s role [5, 8]. Recent guidance by NICE states there should be a designated healthcare professional that carries overall responsibility for the process and it should be undertaken by trained, competent health professionals—ideally pharmacists, pharmacy technicians, nurses or doctors [6]. This study explores the role of each staff member, with a principal focus on the pharmacy team (both pharmacists and pharmacy technicians) and the variability of the procedure between organisations providing secondary care mental health services.

Primary care is the usual first point of contact for healthcare within the NHS and includes GP services. Secondary care refers to specialist services, which are usually accessed following a referral from primary care. Secondary care mental health services include in-patient wards and community teams, such as crisis and home treatment teams, which typically provide intensive support, in the community, for people suffering from a mental health crisis.

People with mental health problems may be particularly susceptible to medicine reconciliation discrepancies due to their highly complicated conditions and drug regimes, as well as the impact of cognitive impairment [8–10]. The pharmacy team has a key role in reducing reconciliation errors within hospital and mental health settings [5, 10, 11]. Very few studies have analysed the process of medicines reconciliation at admission in mental health organisations [5, 12]. Data collected will aid healthcare providers and patients in understanding how the service is delivered by organisations providing secondary mental health care and the role of pharmacists and pharmacy technicians. Medicines reconciliation has been proven to reduce drug regime discrepancies in prior studies [5, 10, 11], and ensuring that the process is undertaken regularly and fully will benefit patient safety.

## Aim

To investigate how medicines reconciliation is delivered within organisations providing mental health services across England, Northern Ireland, Scotland and Wales.

The objectives were:

1. To identify how and when medicines reconciliation is undertaken within various mental health organisations as well as the role of each staff member in the process and novel approaches to support prospective guidance.
2. To identify the level at which organisations providing secondary care mental health services are compliant with NICE guidance [5].

## Ethical approval

Ethical approval from Aston University’s research and ethics committee (pharmacy sub-committee) was obtained.

## Methods

Medicines reconciliation procedures were thoroughly researched using published literature in order to construct appropriate questions for the online self-completion web survey. Questions were discussed and developed between all authors. Questions were then loaded onto the web via Bristol Online Surveys (BOS). The survey was divided into sections pertaining to the respondent, their trust, the medicines reconciliation process, the role of pharmacy, medicines reconciliation on transfer and discharge, targets, discrepancies and an area for further comments respondents would like to make. Each part had a series of relevant open and closed questions in a variety of styles including: single one best answer multiple choice, multiple answer multiple choice, free-text multiple line format questions and ranking questions (Appendix of Supplementary material). One question was displayed per page.

Upon loading the survey online, a pilot test was sent out. A chief, directorate and lead mental health pharmacist from three different mental health trusts, either from England or Wales piloted the survey. The final survey incorporated the changes as suggested from the pilot work.

The survey was then launched to chief pharmacists (or equivalent) in organisations providing secondary care mental health services via BOS in 2014. An email highlighting the purpose of the project and a link directing applicants to the BOS website displaying the survey was sent to eight senior or chief pharmacists that had volunteered from each region of the UK (North, East, South and West England, London, Scotland, Wales, and Northern Ireland). They then forwarded this email to chief pharmacists or professionals of equivalent status in all mental health organisations within their region. Volunteer senior or chief pharmacists were used in preference to emailing organisations directly from an unknown student, in an attempt to improve the response rate. Additionally, the email and survey link was sent to the CMHP email discussion group, to remind the participants about the study and thus also to improve the response rate. A list of organisations providing mental health services within each region was obtained online in order to keep record of responses and determine a response rate [13–17].

The survey was open for 4 weeks; participants were sent weekly reminder emails from the eight volunteer senior or chief pharmacists from each region, members of the CMHP email discussion group were also sent weekly reminder emails. Participants were able to save and complete the survey at a later time whilst the survey was open.

The following definition of medicines reconciliation was set to aid participants in completing the survey questions:

Medicines reconciliation is the process of identifying and maintaining an accurate list of a patient’s current medications (including name, dosage, frequency, and route). A minimum of two sources must be used to confirm the medication a patient was prescribed prior to admission. E.g. from the GP surgery and carer. The process is only complete if no discrepancies are found or when discrepancies found are resolved [5].

Throughout the data-collection stage, a log of each respondent’s answers was obtained via the BOS programme allowing for the full data set to be obtained on closure of the survey. BOS automatically calculates statistical values including the mean, confidence intervals, lower and upper quartiles for each question in the survey. Quantitative data was analysed using descriptive statistics, whilst qualitative data involved scrutinising all free-text answers for common themes contributing to a justification for results [18].

## Results

### Overview

Only one response from each organisation was required. However, in two cases two chief pharmacists (or equivalent) each completed the survey. These responses were compared to each other, and as the answers were broadly similar the answers from the participant rendering more detail throughout his/her answers were included. The other two results were discarded confidentially. As two sets of results were taken out from the data set, all statistical measures had to be re-calculated by hand.

Basic descriptive statistics were used to quantify the percentages of persons providing specific answers. A total of 44 responses were obtained, however only 42 were used in analysis due to the multiple surveys received from two organisations. The response received by each country and their corresponding response rates are shown in Table 1 below. The overall response rate is 51.9 %.

**Table 1:** Response rate to survey

	England	Scotland	Wales	Northern Ireland	Total
Number of mental health organisations that responded (percentage out of total responses)	31 (73.8 %)	9 (21.4 %)	2 (4.7 %)	0 (0 %)	42
Response rate per region	55.4 % (31 of 56)	64.3 % (9 of 14)	33.3 % (2 of 6)	0 % (0 of 5)	51.9 % (42 of 81)

The first row represents the number of mental health organisations within each region that completed the survey and the percentage that region makes up for out of total responses. The second row represents the response rates within each region surveyed as well as the total response rate of all regions combined

### Formal policies and protocols

**Fig. 1:**
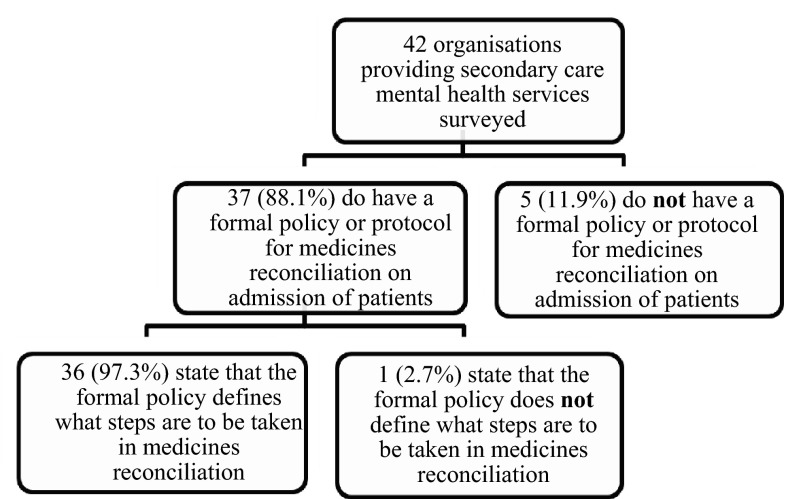
Number of mental health organisations with policies and procedures

Thirty-seven of the mental health organisations (88.1 %) have a formal policy or protocol for medicines reconciliation on admission of patients, whilst five (11.9 %) do not. Of those with a formal policy or protocol for medicines reconciliation, 36 organisations (97.3 %) state that the formal policy also defined what steps are to be taken in medicines reconciliation (Fig. 1).

### Time frames

Thirty-nine (92.9 %) organisations reported they have a time scale in which to complete reconciliations. Of the 39 organisations with a target time frame, 18 organisations (46.2 %) aim for completing medicines reconciliation within 24 h, 3 more organisations (total 21 organisations, 53.8 %) within 48 h, 17 more organisations (total 38 organisations, 97.4 %) within 72 h and one more organisation (total 39 organisations, 100 %) within 1 week. 77.1 % of mental health organisations estimate they complete medicines reconciliation by 5 pm the day following admission. In 24, 48, 72 h and 1 week following admission; 79.3, 84.6, 89.8 and 97.6 % of mental health organisations estimate medicines reconciliations to be achieved respectively (Table 2).

**Table 2:** Time frames

Time frame	Number of organisations aiming for the time frame (percentage aiming for the indicated time frame or shorter)	Percentage of organisations reaching the time frame (completing medicines reconciliation within the time frame) (%)
5 pm the day after admission	0 %	77.1
24 h	18 (46.2 %)	79.3
48 h	3 (53.8 %)	84.6
72 h	17 (97.4 %)	89.8
Up to 1 week	1 (100 %)	97.6

The second column represents each organisations target time frame for medicines reconciliation to be completed in their policy. The percentage figures in this column represent the percentage of organisations aiming for the indicated target or shorter time frame. The final column is estimated percentages of organisations completing medicines reconciliations within the specified time frame from survey results

### Acute, non-acute admission wards and teams admitting from primary care

Thirty respondents (71.4 %) said members of pharmacy staff carry out medicines reconciliation daily (excluding weekends and bank holidays) on acute admission wards. Of the 12 organisations (28.6 %) that do not carry out medicines reconciliation daily on acute admission wards, five organisations (41.7 %) indicated the service is delivered once per week or less on acute admission wards, 2 organisations (16.7 %) twice per week, 4 organisations (33.3 %) three times per week and one organisation (8.3 %) four times per week. Participants were also asked if any members of pharmacy staff carry out medicines reconciliation for any other teams that admit patients from primary care (e.g. crisis teams, home treatment teams). Sixteen organisations (38.1 %) answered yes; data on frequency was not obtained.

A total of 39 organisations reported pharmacy members carry out medicines reconciliation on non-acute admission wards compared to three organisations answering ‘not applicable’ to the survey question. Of the 39 organisations, seventeen organisations (40.5 %) said pharmacy members carry out medicines reconciliation daily (excluding weekends and bank holidays). Of the remaining 22 organisations (52.4 %) not completing it daily, 14 organisations (63.6 %) said it is done once per week or less on non-acute admission wards, 4 organisations (18.2 %) twice per week and 4 organisations (18.2 %) three times per week.

### Medicines reconciliation on transfer

Twenty-two organisations (52.4 %) do not undertake medicines reconciliation when patients are moved internally within their trust. Free-text answers reveal medicines reconciliation on transfer differs from admission because it is a “quick check that it has been done on admission, all changes since admission are noted and clear.”

### Staff roles

95.2 % of responses indicated pharmacists were accountable for delivering medicines reconciliation and 71.4 % pharmacy technicians involved in the process. Doctors and nurses were 66.7 and 42.9 % respectively accountable for undertaking the procedure.

### Staff training

**Fig. 2:**
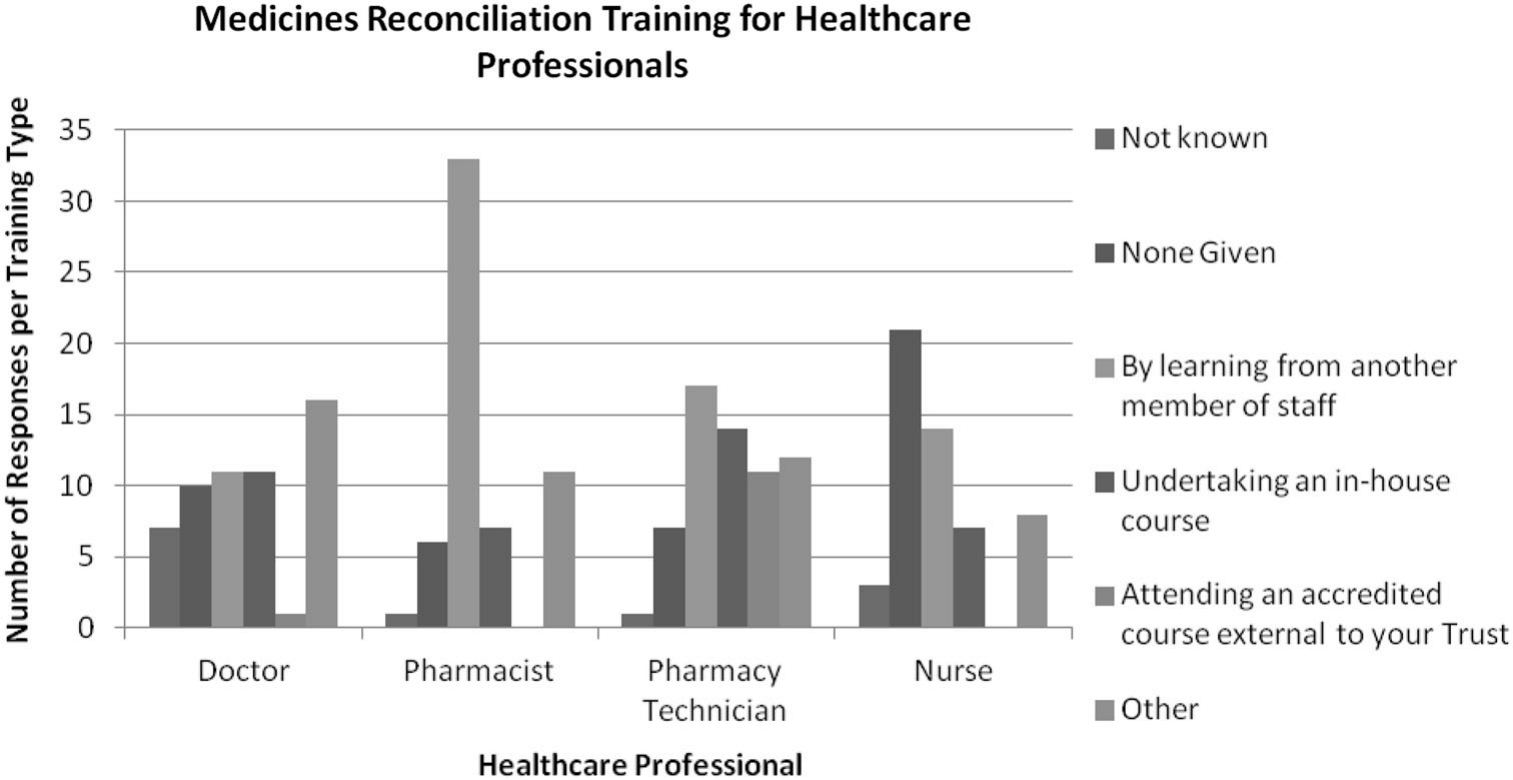
Medicines reconciliation training for healthcare professionals. Response totals are above 42 as more than one option could be selected by the respondent

Respondents were asked to indicate what type of training each healthcare professional receives (Fig. 2). Sixteen organisations selected ‘other’ for doctors. Free-text answers indicate “doctors are trained during induction by the pharmacy team.” Thirty-three organisations and 17 organisations respectively indicated pharmacists and pharmacy technicians are trained by learning from another member of staff. Free-text answers in response to ‘other’ methods included “supervised or observed assessment.” Results indicate the pharmacy team usually learns via another staff member and they are not receiving any formal form of training or assessment before completing medicines reconciliation or training doctors. Four organisations indicated either close supervision or observed assessment for pharmacists. A total of 26 organisations said pharmacy technicians complete an in-house course or attend an accredited course external to their organisation whereas seven organisations have pharmacists undertake an in-house course and none are sent for an external course.

## Discussion

Almost 80 % of organisations appear to be completing medicines reconciliation, on their acute admission wards, within 24 h of admission in line with NICE and WHO guidance [5–7], however only 46.2 % set a target of having the process completed within 24 h. The reasons for these variations are not clear. Supporting organisations to aim for a higher percentage within 24 h may be practical and could be very beneficial to the system. Achieving faster rates can be encouraged via Commissioning for Quality and Innovation (CQUIN) payment framework, contractual performance measures, Key Performance Indicators (KPI) targets or the Scottish Patient Safety Programme for Mental Health.

Establishments that do not have daily pharmacy visits and low reconciliation levels throughout the week should consider improvements in the medicines reconciliation process. Some mental health organisations that are unable to have daily pharmacist visits to every ward had lower reconciliation levels; however it did not show a direct correlation through this study’s results.

Generally, pharmacy staff is not conducting medicines reconciliation for other units that admit from primary care (such as crisis and home treatment teams). The recent NICE guidance highlighted the need for medicines reconciliation when patients are transferred between wards [6]. This research found that medicines reconciliation on transfer is generally much quicker and less exhaustive than on admission. It “is not as rigorous, it’s an accuracy check of any changes since admission rather than a full reconciliation of the entire medication history.” However, the care is still within the mental health organisation; hence the responsibility continues to lie within the organisation providing the care.

Both NICE and WHO highlight the importance of staff training [6, 7]. The lack of formal training, particularly for pharmacy staff, may raise concerns that medicines reconciliation is not creating an environment of clinical excellence. However, it must be noted that many of these organisations have introduced medicines reconciliation without any extra funding.

Earlier research has identified that medication errors are common when people with mental health problems move between primary and secondary care mental health services [8, 19, 20]. Previous studies in the UK and America have confirmed medicines reconciliation is effective in reducing medication errors within hospital admission wards, emergency departments and mental health trusts [8, 11, 12]. A recent audit-based quality improvement programme had focussed on medicines reconciliation on admission to in-patient psychiatric care in the UK. The audit had data submitted for 1790 patients from 42 trusts at baseline and 2296 patients from 43 trusts at re-audit (16 months later) [21]. Like our research, the audit found most medicine reconciliations were conducted by pharmacy staff and occurred within 24 h of admission [21]. Specifically, the audit found that at baseline and at re-audit, pharmacy staff (pharmacist or pharmacy technician) were involved in 1251 (70 %) and 1902 (83 %) of patients’ medicine reconciliations respectively [21].

This research found that most organisations do not have members of the pharmacy team reconciling medication on units other than acute admission wards, for example crisis teams that admit directly from primary care. This is in contrast with acute admission wards. These other units are an increasingly important link between primary and secondary care, and it is vital that medicines reconciliation services are delivered regularly and accurately to these units, as well as to acute admission wards. Organisations may need to focus on enhancing this service within these units to improve patient safety. Mental health organisations should also consider introducing formal training programmes for pharmacy staff involved in medicines reconciliation perhaps as part of the pharmacy department’s training programme.

Further research should investigate the actual level of medicines reconciliation, in contrast to self-reported levels, delivered by both pharmacy staff and other clinicians to all units that admit from primary care. Work should also focus on developing the role of pharmacy in medicines reconciliation within other units that admit from primary care and during transfer between units within the organisation, as well as the impact of such services on medication safety and other key clinical outcomes. Finally, validated training programmes for pharmacy staff should be developed.

Limitations of the study include:

1. The overall response rate was 51.9 % therefore the results should be interpreted with some caution.
2. All responses were self-reported by respondents and the study did not aim to identity the accurate level of services.
3. The survey focussed on services delivered by members of the pharmacy team.
4. No responses were obtained from Northern Ireland; therefore the research cannot necessarily be extrapolated to the whole of the UK.
5. The survey did not question the rate of medicine reconciliations being carried out throughout the weekend and any holidays. However with lower staff numbers on weekends and holidays, it is likely the rate of reconciliations would be low which could be hazardous to patient health safety.
6. In survey questions asking how often medicines reconciliation is carried out on acute and non-acute admission wards, there was no option to select “zero” times per week. Majority of respondents selected the once weekly option, however it may be possible that the better suited answer would have been zero times per week for these mental health organisations and there was no way to specify this.

## Conclusions

Numerous studies have been completed within hospitals on medicines reconciliation, however this study is one of few concerning organisations providing secondary care mental health services within the UK. This survey study has established a background to how medicines reconciliation is currently delivered in mental health wards and has raised some areas of concern in which additional research should be carried out. Overall, NICE guidance is relatively well implemented as nearly 80 % of mental health organisations report conducting medicines reconciliation within 24 h of admission on acute admission wards (excluding weekends and holidays). However, increasingly people with mental health problems are admitted to secondary organisations via crisis or home treatment teams and only 38 % of organisations self-report to reconciling the medicines of these patients.

The impact of current staff training procedures and how well they adhere to best practice without formal training, suggests pharmacy departments need to consider formal training and competency assessments for the pharmacy team. As results indicated medicines reconciliation on transfer is rarely completed and when it is, the process significantly differs to that on admission; secondary care mental health organisations should consider appropriate guidelines for transfer and whether a full reconciliation of medicines is compulsory or not.

## Electronic supplementary material

Below is the link to the electronic supplementary material.

Supplementary material 1 (DOCX 22 kb)
