# Supplementary material for: Medicines reconciliation in comparison with NICE guidelines across secondary care mental health organisations
Source: Int J Clin Pharm. 2016 Jan 6;38:289–95. doi: 10.1007/s11096-015-0236-7 (PMC4828472; doi:10.1007/s11096-015-0236-7)
Supplement: Supplementary file 1 — Supplementary material 1 (DOCX 22 kb) [file 11096_2015_236_MOESM1_ESM.docx]

**Appendix 1**

**Self-Completion Web Survey Questions Evaluating Medicines Reconciliation Service Levels across Mental Health Organisations in the UK**

**Section 1: Welcome**

Thank you for taking the time to complete this survey. Your feedback is important in improving medicines reconciliation services delivered within Mental Health Organisations to patients in the NHS. This survey aims to understand the process of medicines reconciliation across the United Kingdom and highlight the pharmacy team’s involvement.

All data collected in this survey will be held securely and only used for the purpose of this study. All responses will be kept confidential and all identifiers will be removed. The project has been approved by the Aston Research Ethics Committee. Cookies stored by your web browser will not be used in this survey.

Results will be presented at the annual College of Mental Health Pharmacy Conference via poster or presentation.

This survey should take approximately 20 to 30 minutes to complete.

*In order to progress through this survey, please click the Continue button to proceed to the next page.*

**Please note that all questions are mandatory and once you have clicked on the CONTINUE button, your answers are submitted and you cannot return to review or amend that page.**

**Section 2: About You**

1. What is your name?
2. What is your job title?

**Section 3: About your Trust (or equivalent)**

1. Where is your Trust located?
   - England
   - Wales
   - Scotland
   - Northern Ireland
2. What is the name of your Trust?
3. How many psychiatric ward admission beds does your Trust have?

**Section 4-8: About the Medicines Reconciliation Process**

Please use the following definition of medicines reconciliation to complete the questions below.

*Medicines reconciliation is the process of identifying and maintaining an accurate list of a patient’s current medications (including name, dosage, frequency, and route). A minimum of two sources must be used to confirm the medication a patient was prescribed prior to admission. E.g. from the GP surgery and carer. The process is only complete if no discrepancies are found or when discrepancies found are resolved^5^.*

1. Does your Trust and/or mental health wards have a formal policy or protocol for medicines reconciliation on admission of patients to hospital? YES/NO

- If yes, does the formal policy or protocol define what steps are taken in the medicines reconciliation process?
  - If yes, please select which of the following steps are included and add any additional steps your policy includes.
    - - Checking the patients drug history
      - Confirming the patients drug history with two sources
      - Raising any discrepancies with the prescriber
      - Resolving these discrepancies with the prescriber
      - Other (*please specify)*

1. Which of the following types of staff undertake medicines reconciliation as defined above. If yes, please estimate the percentage of the total medicines reconciliations done by the corresponding professional.
   - Doctors YES/NO
     - Percentage: _____

- Pharmacists YES/NO
  - Percentage: _____
- Pharmacy technicians YES/NO
  - Percentage: _____
- Nurses YES/NO
  - Percentage: _____
- If necessary, please comment on any of the above percentages (e.g. if pharmacists and pharmacy technicians work together, give a combined percentage of the medicines reconciliation they both do).

1. Are there any other members of staff whom undertake medicines reconciliation as defined above? YES/NO
   - If yes, please specify which other members of staff undertake medicines reconciliation and estimate the percentage of the total medicines reconciliations done by these staff members.
2. What training is provided to **doctors** undertaking medicines reconciliation? *(select all that apply)*
   - Not known
   - None given
   - By learning from another member of staff
   - Undertaking an in-house course
   - Attending an accredited course external to your Trust
   - Other *(please specify)*
3. What training is provided to **pharmacists** undertaking medicines reconciliation? *(select all that apply)*
   - Not known
   - None given
   - By learning from another member of staff
   - Undertaking an in-house course
   - Attending an accredited course external to your Trust
   - Other *(please specify)*
4. What training is provided to **pharmacy technicians** undertaking medicines reconciliation? *(select all that apply)*
   - Not known
   - None given
   - By learning from another member of staff
   - Undertaking an in-house course
   - Attending an accredited course external to your Trust
   - Other *(please specify)*
5. What training is provided to **nurses** undertaking medicines reconciliation? *(select all that apply)*
   - Not known
   - None given
   - By learning from another member of staff
   - Undertaking an in-house course
   - Attending an accredited course external to your Trust
   - Other *(please specify)*
6. Does your Trust aim to complete medicines reconciliation within a defined time frame? YES/NO
   - If yes, what is the time scale?
7. Using the above definition of medicines reconciliation, are you able to put a percentage on how many patients will have had a medicines reconciliation completed by 5pm the day after admission? YES/NO
   - If yes, please estimate the percentage.
   - If no, please estimate a percentage figure for completions and define this against a criteria (e.g. 80% completed within 2 working days).

**Section 9 – 12: Role of Pharmacy in Medicines Reconciliation**

1. Do members of pharmacy staff carry out medicines reconciliation daily (excluding weekends and bank holidays) on acute admission wards? YES/NO
   - If it is **not** done daily, how many days a week is medicine reconciliation done on *only* acute admission wards? 1, 2, 3, 4, 5
2. Do members of pharmacy staff carry out medicines reconciliation daily (excluding weekends and bank holidays) on non-acute admission wards (if any exist)? YES/NO/NOT APPLICABLE
   - If it is **not** done daily, how many days a week is medicines reconciliation done on the non-acute admission wards? 1, 2, 3, 4, 5
3. Please estimate the percentage of **all** admissions in which members of the pharmacy team reconcile medicines (even if it is not completed within the anticipated target time scale).

- <10%
- 10%-20%
- 20%-30%
- 30%-40%
- 40%-50%
- 50%-60%
- 60%-70%
- 70%-80%
- 80%-90%
- >90%

1. How do pharmacy staff members check patient records when conducting medicines reconciliations? *(select all that apply)*

- Refer to patient’s own drugs (PODs)
- Contact GP surgery via fax
- Contact GP surgery via telephone and speaking to reception
- Ask the patient where appropriate
- Ask family where appropriate
- Refer to historical notes
- Refer to an electronic patient record (e.g. RiO, PARIS, JAC)
- Contact the patients community pharmacy
- Contact community mental health services
- Contact drug and alcohol services
- Contact other healthcare organisations
- Refer to summary care records (also see question 19)
- Other *(please specify)*

1. Does your Trust currently use Summary Care Records (SCR)? YES/NO

- If yes, for how long has your Trust been using SCRs?
- If no, are they currently being piloted in your Trust?

1. Do any members of pharmacy staff conduct medicines reconciliations for any other teams that admit patients from primary care (e.g. crisis teams, home treatment teams)? YES/NO
   1. If yes, estimate the percentage of all admissions to other teams (e.g. crisis) in which members of the pharmacy team reconcile medicines.

- <10%
- 10%-20%
- 20%-30%
- 30%-40%
- 40%-50%
- 50%-60%
- 60%-70%
- 70%-80%
- 80%-90%
- >90

**Section 13-14: Medicines Reconciliation on Transfer and Discharge**

1. Is medicines reconciliation undertaken when patients are moved internally within your Trust (i.e. between different teams)? YES/NO

- If yes, is the medicines reconciliation process the same to that upon admission? YES/NO
- If no, please describe the altered process.

1. Is medicines reconciliation undertaken on discharge? YES/NO/IN SOME UNITS

- If no, is your Trust piloting this in some areas? YES/NO

1. If medicines reconciliation is undertaken on discharge, is the process the same as that upon admission? YES/NO/NOT APPLICABLE
   1. If no, please describe the altered process.
2. If medicines reconciliation is undertaken on discharge in some units, is the process the same as that upon admission? YES/NO/NOT APPLICABLE
   1. Please indicate in which units medicines reconciliation is undertaken on discharge and describe the altered process? If not applicable, please type *N/A* and click **Continue** to proceed to the next page.

**Section 15: Medicines Reconciliation Targets**

1. Does your Trust have a Commissioning for Quality and Innovation (CQUIN) target or contractual performance measure for medicines reconciliation? YES/NO/NOT APPLICABLE
   1. If yes, please indicate whether your Trust has a CQUIN target or contractual performance measure and what the target/measure is?

**Section 16: Medicines Reconciliation Discrepancies**

1. According to your opinion, please rank the following medicines reconciliation discrepancies (taking into account admission, transfer and discharge), from most to least common for your Trust. [1=most common; 7=least common]

- Omission (medicine that should have been prescribed has not been prescribed)
- Wrong/unclear dosage
- Wrong/unclear strength
- Wrong/unclear formulation
- Wrong/unclear frequency
- Wrong/unclear route of administration
- Additional medication (medication that should not have been prescribed, is prescribed)

**Section 17: Further Information**

1. Please give any further information or comments regarding issues relating to medicines reconciliation in your Trust that will support your answers to this survey. Feel free to also include any feedback for this survey. *(Optional)*

**Thank you for taking the time to complete this survey.**

Your answers will be held securely and only used for the purpose of this study. All responses will be kept confidential and all identifiers will be removed.

Results will be presented at the annual College of Mental Health Pharmacy Conference.
